# Dark Tetrad personality traits, paraphilic interests, and the role of impulsivity: an EEG-study using a Go/No-Go paradigm

**DOI:** 10.1038/s41598-024-61214-z

**Published:** 2024-05-13

**Authors:** Maria M. Lassche, Luca Lasogga, Melissa S. de Roos, Amber Leeflang, Vanesa Ajazi, Magda Axioti, Eric Rassin, Josanne D. M. van Dongen

**Affiliations:** 1 Department of Psychology, Education and Child Studies, Erasmus University Rotterdam, Burg. Oudlaan 50, 3062 PA Rotterdam, The Netherlands; 2 Department of Psychiatry, Psychotherapy and Psychosomatics, RWTH Aachen, Aachen, Germany; 3 Department of General Psychology, University of Padua, Padua, Italy

**Keywords:** Dark Tetrad, Paraphilia, Impulsivity, Response inhibition, P3, Human behaviour, Neuroscience

## Abstract

Maladaptive personality traits, such as ‘dark personalities’ are found to result in a diverse set of negative outcomes, including paraphilic interests and associated (illegal) behaviors. It is however unclear how these are exactly related, and if related, if then only those individuals higher on dark personality traits and higher impulsivity engage in paraphilic behaviors. In the current study, 50 participants were recruited to investigate the relationship between Dark Tetrad personality traits (i.e., narcissism, psychopathy, Machiavellianism and everyday sadism), paraphilic interests (arousal and behavior) and the moderating role of impulsivity. Personality and paraphilic interests were investigated through self-report questionnaires. Impulsivity was measured both through self-reported dysfunctional impulsivity and the P3 event related potential using electroencephalography during the Go/No-Go task (i.e. response inhibition). The results showed that there was a positive association between psychopathy, sadism and paraphilic interests. Whereas everyday sadism was associated with paraphilic (self-reported) arousal, psychopathy was associated with paraphilic behavior. Although P3 amplitude was not associated with paraphilic interests, self-reported dysfunctional impulsivity was associated with paraphilic behavior specifically. However, there was no moderating role of dysfunctional impulsivity and response inhibition (P3) in the relationship between psychopathy and paraphilic behavior. Findings indicate that the relation between specific dark personalities and paraphilic interests may be more complex than initially thought. Nevertheless, risk assessment and intervention approaches for paraphilia and related behavior both may benefit from incorporating Dark Tetrad and impulsivity measurements.

Sexual interests that deviate from societal norms may be more prevalent than previously thought^1^. Such deviant, or paraphilic interests, are mostly not acted upon^2^. Even when people do engage in paraphilic behavior, this typically occurs in a consenting context^3^. However, at the extreme end of the spectrum, paraphilic interest could be linked to an increased risk for engaging in sexually aggressive, coercive (illegal) activities that may have adverse, harmful social consequences^4,5^. Given that this interest is dimensional rather than categorical^6^, and most research has been conducted in clinical settings, it is important to examine paraphilic interest and behavior in the general population.

## Dark Tetrad personality traits and deviant sexual interests and behaviors

Previously, the Dark Triad comprised three personality traits (e.g., Machiavellianism, narcissism, and psychopathy) associated with socially malevolent characteristics^7^. Recently, the personality trait everyday sadism was added to the former three traits, resulting in the Dark Tetrad^8,9^. These maladaptive personality traits have been linked to adverse outcomes such as stalking, sexual harassment, and sexual coercion^10^. In addition, various studies have demonstrated that Dark Tetrad traits are linked to an increased likelihood of developing paraphilic interests and behaviors^11^. The four constructs share characteristics of manipulation, callousness, lack of empathy and deceitful behavior^8^, but also contain unique features that seem to be distinctively related to paraphilic interests and behaviors^12,13^.

Psychopathy is characterized by a lack of remorse and empathy, reduced self-control, emotional control, and impulsive, anti-social behavior^14^. This general psychopathic personality trait is found to be most frequently associated with paraphilic interests and subsequent sexually aggressive, coercive behaviors^15,16^. For instance, research has found that a combination of psychopathic traits such as affective facets, but mostly antisociality, are related to sexual violence and sexual sadism^17,18^. Everyday sadism is regarded as a subclinical personality construct of enjoying cruelty, humiliation and violent behavior and taking pleasure from other people’s psychological or physical suffering^8,19^. Moreover, everyday sadism strongly predicts higher sexual drive^9^ and is associated with increased engagement in harmful, sexually aggressive behaviors^16,20^. Although everyday sadism is linked to paraphilic interest and behaviors, research on its association with paraphilia remains scarce, since everyday sadism is a relatively new construct within the dark personalities literature.

In contrast to psychopathy and everyday sadism, the Dark Tetrad constructs of narcissism and Machiavellianism do not seem to be as strongly related to paraphilic interests and behaviors. Narcissism is marked by either inflated or reduced self-esteem, grandiosity and a sense of personal entitlement^14,15^. Although some studies have linked narcissistic traits to sexually aggressive behavior^21^, especially when those individuals are denied access to individuals they desire, others reported narcissism to be associated with less socially aversive and non-aggressive sexual preferences and behaviors^22^. Finally, Machiavellianism involves manipulation, power and strategic planning, whereby individuals high on those traits often engage in controlled, strategic ways of sexual behavior^22^.

In short, the strong relation between Dark Tetrad personalities and sexual deviance seems to hold particularly for psychopathy and everyday sadism. This suggests that the unique features related to those Dark Tetrad components might make individuals vulnerable to develop sexual deviant interests and behaviors.

## Impulsivity, Dark Tetrad and paraphilia

To regulate and inhibit sexual impulses, self-control abilities are crucial^23^. Hereby, impaired self-control might result in impulsivity, which is indicated to facilitate acting upon paraphilic fantasies and engagement in paraphilic behaviors^24^.

Regarding the Dark Tetrad personality traits, both psychopathy and everyday sadism are found to be associated with greater impulsiveness^25^, although see the arguments of Poythress and Hall^26^, who argue that this notion should be revised^1^. According to Jones and Paulhus^27^, the reduced self-control and antisocial behavior related to psychopathy are compatible with dysfunctional impulsivity, which refers to the maladaptive tendency to act without control or forethought for negative consequences of one’s behavior^28,29^. Hereby, impulsivity might impair psychopathic individuals to control their strong sexual drive, resulting in hypersexual behavior^25^. Combined with a lack of remorse and empathy, psychopathic individuals are more willing to lower their standards and use aggressive tactics to obtain sex, without regard for the adverse, social consequences^27,30–32^. As such, paraphilic behavior associated with psychopathy may be the result of distorted inhibition by negative emotions of others, which predisposes psychopathic individuals to easily engage in sexually coercive, aggressive behaviors^27,32^. In other words, psychopathic personality might only be related to paraphilic engagement (i.e. behaviors) in individuals who have less self-control, or are impulsive.

Regarding the relation between sadism and paraphilic behaviors, researchers have suggested that paraphilic behaviors are the result of experiencing pleasure from perceiving suffering or pain in others, instead of deficits in inhibition^27,32^. However, sadistic preferences in isolation are not sufficient for engagement in sexual deviant behavior, as many individuals who endorse sexually sadistic fantasies do not engage in violent, aggressive sexual activities^28,33^. Rather, the combination of sadistic preferences with reduced self-control might be necessary for sadistic sexual offenses to occur^34^.

In contrast to psychopathy and everyday sadism, Machiavellianism and narcissism are associated with either increased self-control or functional impulsivity (e.g., extraversion), in an attempt to gain social benefits^27^. This differential association between the Dark Tetrad constructs and impulsivity might explain the distinct prevalence of sexual deviant behaviors in each construct^35^.

## Response inhibition

An important aspect related to impulsivity is the ability to inhibit prepotent (automatic) behavioral responses, referred to as *response inhibition*^36^*.* A commonly used task to investigate response inhibition is the Go/No-Go task, which requires participants to rapidly respond to Go-stimuli, whereas participants must withhold such response to No-Go stimuli^37^. Hereby, impaired inhibition during No-Go trials of the Go/No-Go task has been linked to increased levels of impulsivity in healthy volunteers^38^ and in clinical samples^39^.

Various studies have indicated that participants with psychopathy demonstrate reduced response inhibition during No-Go trials, compared to non-psychopathic participants^40,41^. Hereby, the impaired inhibition observed in psychopathic individuals during the Go/No-Go task was found to be particularly related to psychopathy traits associated with an impulsive lifestyle^41^.

A common method to measure neurocognitive mechanisms, such as response inhibition is the electroencephalogram (EEG) during the Go/No-Go task^42^. While responding to No-Go trials, frontal areas exhibit different indices of response inhibition in the form of event related potentials (ERP). Two main ERPs that have been frequently used to measure response inhibition are the P3, which is peaking 300–600 ms after No-Go stimulus onset, and the N2, which occurs 250–350 ms after stimulus onset^42,43^. The idea behind this is that No-Go trials are more cognitively demanding, since inhibition of an automatic response is required. As a result, inhibitory-related frontal brain areas become more active and are suggested to generate the elevated P3 and N2 amplitudes during No-Go trials^43^. However, according to previous research the P3 index is a more valid indicator of response inhibition during no-go trials compared to N2. Whereas N2 was found to be related to response conflict, P3 was suggested to be related to response inhibition^44,45^.

In addition, previous research found differences in P3 rather than N2 deflections when investigating the relationship between response inhibition and psychopathy. P3 amplitudes were demonstrated to be reduced in psychopathic participants during No-Go trials, when compared to healthy, non-psychopathic participants^46^. These findings support the idea that the disinhibited behavior observed in psychopaths may be the result of abnormal executive functioning in inhibitory-related frontal brain areas. However, other studies reported no differences^47^ or rather enhanced P3 amplitude in psychopathic individuals during No-Go trials, when compared to healthy individuals^48^. Hence, the neuroscientific findings on the role of cognitive inhibition in relation to the disinhibited behavior in psychopaths are inconsistent. Therefore, the exact role of impaired response inhibition in relation to psychopathy remains unclear.

In relation to paraphilia, deficits in executive functioning also seem to result in sexually deviant behavior, due to the failure to control and inhibit sexual urges^49^. Research in forensic and clinical populations shows that sexually deviant individuals also demonstrate reduced cognitive inhibition during No-Go trials, as compared to healthy participants^49,50^. As such, it is plausible that the impaired cognitive inhibition related to impulsivity traits in psychopathic individuals might also predispose them to engage in paraphilic behavior.

However, the exact role of response inhibition in relation to sexual deviant behavior remains unclear, as some studies failed to find significant differences in (cognitive) response inhibition between sexually deviant and healthy individuals during No-Go trials of the Go/No-Go task^51^.

## Present study

The current study aimed to gain a more elaborate understanding of the relationship between Dark Tetrad personality traits and paraphilic arousal and behaviors. Based on the research findings described above, we expected that the Dark Tetrad construct of psychopathy and everyday sadism would be positively related to paraphilic arousal and the engagement in paraphilic activities (i.e. paraphilic behavior).

Although, previous research has been conducted on impulsivity and its relationship with sexual deviances and dark personality traits separately, the effect of impulsivity on the relationship between the latter variables together has not been studied. As previous research indicated that the psychopathy trait of the Dark Tetrad is mostly associated with impulsivity^52^, we aimed to replicate that finding and subsequently studied whether the association between psychopathy and paraphilic behavior would be moderated by traits of impulsivity. Specifically, we expected that the relationship between psychopathy and paraphilic behavior would be influenced by dysfunctional impulsivity and P3 amplitudes during No-Go trials of the Go/No-Go task.

## Results

### Paraphilic interests, Dark Tetrad, and impulsivity

Descriptive statistics of all continuous variables are presented in Table 1. Table 2 shows Pearson correlations between each of the Dark Tetrad constructs, paraphilic arousal, paraphilic behavior, dysfunctional impulsivity and P3 amplitude. At face value, the highest correlates were those between paraphilic arousal and psychopathic traits and everyday sadism. Although Machiavellianism and Narcissism correlated less strongly with paraphilic arousal, these correlations also reached significance. Only moderate significant correlations were found between psychopathic traits and everyday sadistic traits on the one hand, and paraphilic behavior on the other hand. Regarding the Dark Tetrad constructs, dysfunctional impulsivity traits negatively correlated with all traits except Machiavellianism, indicating that those SD4 personality traits are associated with greater dysfunctional impulsivity Further, although scores on dysfunctional impulsivity traits did not significantly correlate with P3 amplitude (i.e., response inhibition), dysfunctional impulsivity traits negatively correlated with paraphilic behavior, and everyday sadism significantly correlated with P3 amplitude.

**Table 1:** Descriptive statistics of all independent and dependent variables.

Variable	*M*	*SD*	*Min*	*Max*	α
P3 Amplitude	− 1.90	3.36	− 10.74	3.45	
Dysfunctional impulsivity	20.3	3.0	12	24	0.80
Paraphilic behavior	56.8	10.9	39	88	0.78
Paraphilic arousal	93.5	25.0	42	233	0.89
Narcissism	20.5	3.7	11	28	0.61
Machiavellianism	22.6	4.3	16	34	0.72
Psychopathy	15.6	5.0	7	29	0.80
Everyday sadism	16.7	6.0	7	32	0.80
Age	20.98	2.53	18	27

**Table 2:** Pearson correlations between the SD4, the Paraphilias scale and the dysfunctional impulsivity scale of the DII.

Variable	1	2	3	4	5	6	7
1. P3 amplitude							
2. Dysfunctional impulsivity	0.07						
3. Paraphilic behavior	− 0.09	− 0.29*					
4. Paraphilic arousal	− 0.13	− 0.23	0.72**				
5. Narcissism	0.06	− 0.30*	0.26	0.29*			
6. Machiavellianism	0.02	− 0.25	0.22	0.35*	0.32*		
7. Psychopathy	0.05	− 0.43**	0.49**	0.38**	0.50**	0.42**	
8. Everyday sadism	− 0.30*	− 0.42**	0.43**	0.66**	0.41**	0.45**	0.53**

*N* = 50. *p < .05, **p < .01.

### Dark Tetrad traits as predictors of paraphilic interests

To assess whether the Dark Tetrad components of psychopathy and everyday sadism are predictive of paraphilic behavior, a two-stage multiple hierarchical regression was performed. Since, the outcomes of the exploratory correlations indicated that only psychopathy and everyday sadism significantly related with paraphilic behavior, only those Dark Tetrad personality traits were included as predictors into the regression analysis. Psychopathy was entered at step 1 of the analysis as the exploratory correlation analysis revealed that psychopathy most strongly correlated with engagement in paraphilic activities. Then, everyday sadism was entered at step 2 of the analysis to examine its unique contribution to the prediction of engagement in paraphilic activities. The results of the regression analysis are displayed in Table 3. In model 1, psychopathy significantly accounts for 24% of the model. Individuals high on psychopathy traits engaged more often in paraphilic activities than less psychopathic individuals. After adding everyday sadism in model 2, the explained variance increased by 4% adding up to 28% explained variance in paraphilic engagement. However, as can be read from Table 3, the unique contribution of everyday sadism (${\text{R}}_{{{\text{change}}}}^{2}$ = 4%) did not reach significance.

**Table 3:** Results of the Multiple Hierarchical Regression Analysis for Paraphilic Behavior.

	Predictors	*B* [95% BCa CI]	*SE*	*t*	*p*	*R*	R^2	Δ R^2
Model 1						0.49	0.24	0.24
	Psychopathy	1.06 [0.51, 1.61]	0.27	3.89	< 0.001			
Model 2						0.53	0.28	0.04
	Psychopathy	0.79 [0.15, 1.42]	0.32	2.50	< 0.05			
	Everyday sadism	0.43 [− 0.10, 0.96]	0.26	1.63	0.11			

A second regression was conducted to test whether everyday sadism is a stronger predictor for paraphilic arousal compared to psychopathy. Since, the exploratory correlation analysis revealed that everyday sadism most strongly correlated with paraphilic arousal, everyday sadism was included in the first step of the model, which was significant (*F*(1,48) = 8.24, *p* < 0.001). The R-squared for the model was 0.432, indicating that 43.2% of the variance in paraphilic arousal was explained by everyday sadism. In step 2 introducing psychopathy, the model remained significant, *F*(2,47) = 18.04, *p* < 0.000. After including psychopathy, the explained variance increased by 0.4% adding up to 43.4% explained variance in paraphilic arousal. The adjusted R-squared was 0.41, suggesting that the model accounted for 41% of the variance in the paraphilic arousal after adjusting for the level of psychopathy. However, the change in *R*^2^ was not significant, *F*(1,47) = 0.15, *p* = 0.697, indicating that the unique contribution of psychopathy was not significant and only sadism significantly predicted paraphilic arousal. This shows that individuals scoring higher on sadism also scored higher on paraphilic arousal. However, this is not in line with our first hypothesis since we predicted that psychopathy would also be a significant predictor of paraphilic arousal.

### The moderation role of impulsivity

**Figure 1:**
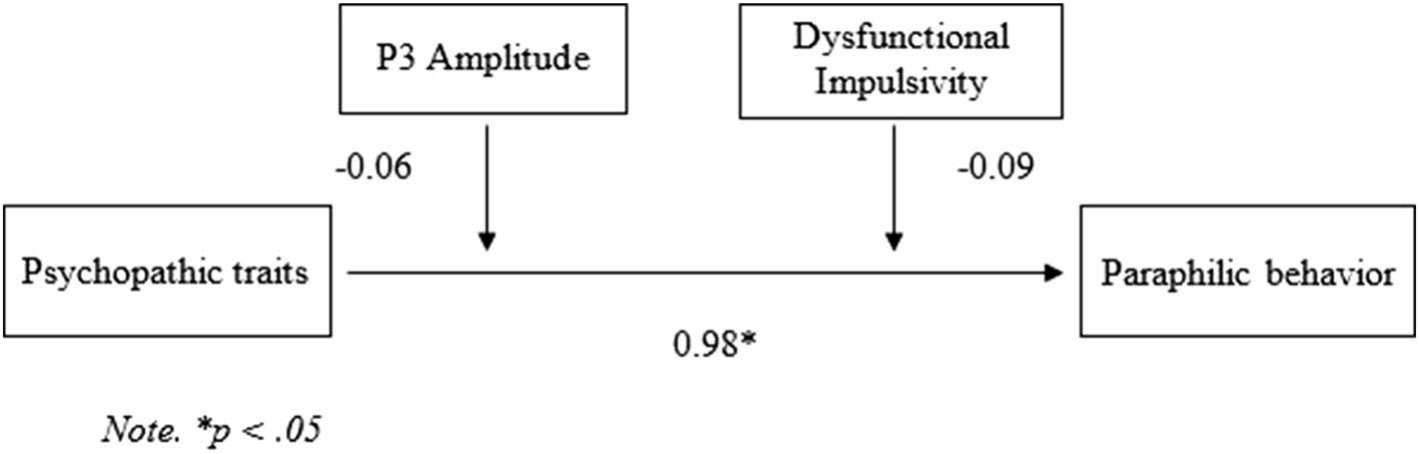
Moderation model for the relation between psychopathic traits and paraphilic behavior moderated by response inhibition (P3) and Dysfunctional Impulsivity.

Finally, a moderation analysis was performed using PROCESS, to investigate the moderating role of impulsivity in the relationship between psychopathy (predictor variable) and paraphilic behavior (outcome variable). In the moderation analysis, P3 amplitude and dysfunctional impulsivity were included as moderator variables. Results of the moderation analysis are displayed in Fig. 1. Contrary to our second hypothesis, we did not find a statistically significant interaction effect of psychopathy and P3 amplitude (*B* = − 0.06, *SE* = 0.11, *p* = 0.611). This outcome suggests that differences in inhibitory control during No-Go trials of the Go/No-Go task do not significantly influence the relationship between psychopathy and paraphilic behavior. Further, against our expectations, we did not find a significant interaction effect of dysfunctional impulsivity and psychopathy (*B* = − 0.09, *SE* = 0.08, *p* = 0.221). This outcome indicates that differences in dysfunctional impulsivity traits do not significantly influence the relationship between psychopathy and paraphilic behavior. In conclusion, results of the moderation analysis indicate that P3 and dysfunctional impulsivity did not serve as a moderator in the relation between psychopathy and paraphilic behavior.

## Discussion

The present study examined the relationship between the Dark Tetrad, paraphilic interests (including both arousal and engagement/behavior) and the moderating role of impulsivity. Partly confirming our first hypothesis and previous research^53^, we found that psychopathy and everyday sadism were most strongly correlated to paraphilic arousal and behavior, when compared to Machiavellianism and narcissism. Although higher levels of Machiavellianism and narcissism were associated with paraphilic arousal, they were not associated with paraphilic behavior. This finding might support the idea that Machiavellian and narcissistic individuals are more prone to engage in more controlled and less deviant sexual behavior^22^.

Although everyday sadism emerged to be significantly associated with paraphilic arousal it was not associated with paraphilic behavior, after controlling for psychopathy. This partly contradicts with previous research that studied sexual aggression and coercion more specifically and found a positive association^20^. Besides the fact that our study examined the broader concept of paraphilic behavior and not aggression and coercion specifically, another explanation for this inconsistent finding may be that Russell and King (2016), distinguished between vicarious, physical and verbal sadism. The Dark Tetrad conceptualizes everyday sadism mainly as its vicarious form. Since Russell and King’s (2016) measure of vicarious sadism was not directly related to sexual aggression and violence this might explain why everyday sadism measured by the Dark Tetrad does not relate to paraphilic behavior in our study. An additional explanation is that previous research has not administered Dark Tetrad traits together but separately. It is advised to measure all Dark Tetrad traits together to account for overlap^9^. However, Russell and King (2016) did not include psychopathy traits to consider the overlap, which might have biased their findings.

Our results further suggest that psychopathic traits may increase the likelihood of engaging in sexually deviant behavior. This finding is in line with a considerable amount of research, relating psychopathy to increased engagement in aggressive, coercive sexual behaviors^22,52^.

Our second hypothesis, that impulsivity would moderate the relation between psychopathic traits and paraphilic engagement was not confirmed. Neither dysfunctional impulsivity nor response inhibition (P3 amplitude) moderated the relation between psychopathic traits and paraphilic behavior. Therefore, our findings suggest that the increased prevalence of paraphilic behaviors in persons scoring higher on psychopathic traits might not necessarily be the result of impaired inhibitory control^54^. One reason for the absent effect might be that paraphilic behavior require some sort of planning^55^, and corroborates with the idea that psychopathic personality is not per definition associated with impulsivity^26^. Paraphilic engagement may also include more affective features of psychopathic personality, instead of those more impulsive-antisocial, as previously shown by Robertson and Knight^22^. Therefore, future studies might want to use other measures of psychopathic personality, that include different factors and facets, to elucidate the different components of psychopathy related to paraphilic engagement.

In addition, our results demonstrate an ambiguous relationship between psychopathy and impulsivity. Similar to findings of Jones and Paulhus^27^, individuals that score higher on psychopathy reported higher levels of dysfunctional impulsivity than individuals scoring less on psychopathic traits. However, no significant impairments in response inhibition, as measured with the P3 during No-Go trials of the Go/No-Go task, were found in relation to psychopathic traits. This result is in line with findings of Munro et al.^47^ and indicates that neural processes involved in response inhibition are not abnormal in psychopathic individuals when both stimuli and context are affectively neutral. Therefore, impulsivity in relation to psychopathy should be considered from a more nuanced perspective.

Interestingly, although our findings do not show an association between everyday sadism and paraphilic behavior, individuals with higher scores on everyday sadism reported greater impulsivity and demonstrated reduced inhibitory control at a neural level during No-Go trials of the Go/No-Go task, when compared to individuals scoring lower on sadism. This study is the first that finds such results. Although everyday sadism was not linked to paraphilic engagement, and we therefore did not examine a moderating effect of impulsivity in such a relation, everyday sadism was found to be associated with paraphilic interests, and therefore future research could further address different forms of impulsivity in relation to everyday sadism and its link to paraphilia. Also, because we especially studied vicarious forms of everyday sadism by using the SD4 in a sample from the general population, this could explain the absence of an association between sadism and paraphilic engagement. Therefore, future studies could examine sadism in clinical and/or offender samples to examine if sadism *is* related to paraphilic engagement in such samples, and whether impulsivity or inhibitory control influences this relation.

Finally, we found that dysfunctional impulsivity was positively associated with the frequency of paraphilic behavior. In other words, individuals higher on dysfunctional impulsivity also engaged more often in paraphilic behavior, supporting the idea that impulsivity might facilitate the engagement in paraphilic behaviors as previously suggested^20^. However, in line with Rosburg et al.^56^, P3 amplitude during No-Go trials was not related to paraphilic behavior. Therefore, the findings challenge the idea that paraphilic behaviors are the result ofw inhibitory impairments, as was suggested by previous studies^48,49^.

Further, since our neurophysiological measure of impulsivity (P3 amplitude) was not related to self-reported impulsivity, different aspects of impulsivity might have been captured by each measurement tool^57^. Hereby, our findings suggest that the different aspects of impulsivity might distinctively relate to paraphilic interests and behaviors. Another explanation might be that our Go/No-Go task was too difficult for the participants, which can be a reason for the absence of disinhibition in paraphilic individuals as previously suggested by Rosburg et al.^56^. In previous studies where paraphilic individuals showed reduced response inhibition, the tasks seemed to be less demanding^53^.

The results of our study should be interpreted with caution for several reasons. Firstly, the relatively small sample size (*N* = 50) enabled us to detect large effects, but less so medium and small effects. Therefore, generalizability might be affected, even more by the relatively homogenous sample consisting mainly of young, high educated white women. Previous research have found that intelligence is differently related to dark personality traits^58^. Future studies should replicate our findings in larger, more diverse samples.

Secondly, the construct of psychopathy was measured as one construct, with no differentiation between separate facets such as interpersonal, lifestyle and affective factors. This approach remains of concern, as impulsivity and response inhibition have been shown to relate to different factors and sub-facets of psychopathy, namely the more impulsive and antisocial subtypes instead of the more ‘primary’ fearless and dominant ones^26,59^ Disregarding sub-facets of psychopathy might explain why no significant relation between psychopathy and response inhibition during the Go/No-Go task was found. Therefore, the use of a dimensional approach including diverse facets regarding psychopathy is recommended for future research.

Thirdly, participants were exclusively confronted with neutral stimuli. As such, we were not able to assess inhibitory control of participants within the context of sexual arousing stimuli specifically. However, to our knowledge, this is the first study to examine the relation between the Dark Tetrad and paraphilias by accounting for inhibitory control mechanisms, including at the neural level. The identified difference in P3 amplitude following the No-Go trials compared to the Go trials in the task indicates that the task in our study was a valid measurement of response inhibition.

Finally, Dark Tetrad personality traits and paraphilic interests and behaviors were measured by self-report questionnaires. Whilst people high on Dark Tetrad traits do not seem prone to giving socially desirable answers in research surveys^60^, asking about a highly personal subject such as sexual interest and behavior might have affected people’s responses.

The implications of the current study are manifold. First, this study identified an association between dark personality traits and paraphilia at a lower end of the spectrum of paraphilic interest. This suggests that even at this lower end, before a potential progression to more extreme interest, personality traits also play a role. Extreme forms of paraphilia could (over time) become problematic, both for the individual and others. Although our findings first have to be replicated in more clinical and/or offender samples, for paraphilias associated with sexual offending behavior (e.g., pedophilia)^61^, an early identification of risk factors might reduce or even prevent future sexual coercive behavior^62^. It might also be a useful focus on intervention for those individuals who struggle with their sexual interests or feel that these are escalating beyond their control. An escalation or loss of control also underlines the importance of impulsivity. Since psychopathic traits and everyday sadism were found to be related to reduced response inhibition and dysfunctional impulsivity, our study results appeal for approaches focusing on improvement of self-control in relation to everyday sadism. However, the exact role of impulsivity and its underlying neurocognitive mechanisms in the relationship between Dark Tetrad traits and paraphilic interests and behaviors remains unclear. Therefore, further research on the neurobiological factors is required to develop effective clinical intervention strategies that help individuals who experience distress due to their sexual interests, as well as those that aim to minimize the risk of engaging in illegal paraphilic behavior, including sexual offending.

In conclusion, everyday sadism has been associated with elevated levels of paraphilic interests, whereas psychopathy was associated with increased engagement in paraphilic behavior specifically. No moderation effect of impulsivity in the relation between psychopathy and paraphilic behaviors was found. However, the results of the present study indicate that dysfunctional impulsivity and response inhibition play a role in both psychopathic personality and everyday sadism and highlights the importance to further examine the role of impulsivity in the development of paraphilic interests.

## Methods

### Participants

The final sample consisted of 50 participants (31 female, 17 male, 2 non-binary) after two participants were excluded, because of too many artefacts in their EEG data. Their age ranged from 18 to 27 years (*M* = 20.98*, SD* = 2.54). A G * Power analysis indicated 48 participants were needed to detect large effects of 0.35 and a statistical power of 0.95, with a regression analysis including a maximum of two predictor variables. The sample consisted mainly of white undergraduate psychology students, who participated for research credit, and a convenience sample from the general population. Participants were excluded based on (1) insufficient knowledge of the English language and (2) the presence of a mental disorder, neurological disorder, or traumatic brain injury. An overview of the sociodemographic characteristics can be found in Table 4.

**Table 4:** Sociodemographic characteristics of participants.

		Frequency	Percent (%)
Sexual orientation	Heterosexual	37	74
	Homosexual	6	12
	Bisexual	6	12
	Asexual	1	2
Country of residence	The Netherlands	45	90
	Germany	1	2
	Spain	1	2
	Vietnam	1	2
	Japan	1	2
	Russia	1	2
Relationship status	Single	20	40
	Dating	3	6
	In a relationship	27	54
Highest level of education	High school diploma	24	48
	College/university, no degree	16	32
	Associate’s degree	1	2
	Bachelor’s degree	7	14
	Master’s degree	2	4
Employment	Employed part-time	10	20
	Unemployed	1	2
	Student	38	76
	Student and part-time worker	1	2
Ethnicity	White	38	76
	Hispanic, Latino or Spanish	2	4
	Black or African American	1	2
	Asian	3	6
	Middle Eastern or North African	1	2
	Mixed	5	10

### Materials

#### Short Dark Tetrad scale

Dark Tetrad personality traits were assessed using the Short Dark Tetrad Scale^9^. The Dark Tetrad is divided into four distinct subscales, with each trait measured by seven items (28 items in total). Participants are asked to what extent they agreed each of the items on a 5-point Likert-type scale applies to them (1 = *strongly disagree,* 5 = *strongly agree*). Sample items include “I really enjoy violent films and violent video games” (sadism). The scale’s internal consistency was high (α = 0.85). Scores were summed, with higher total scores on the subscale indicating higher levels of the trait^9^.

#### Dickman impulsivity inventory

Impulsivity was measured by the second version of the Dickman Impulsivity Inventory (DII)^28^. The DII consists of 23 items related to impulsivity, which participants are asked to answer with either *true* or *false*. The scale consists of 11 items related to functional impulsivity, and 12 items that capture traits related to dysfunctional impulsivity (e.g., “I often get into trouble because I don’t think before I act”). For the purpose of this study, only dysfunctional impulsivity was included. Items were summed with *lower* total scores (i.e. ‘true’) indicating greater dysfunctional impulsivity.

#### Paraphilias scale

The Paraphilias Scale^63^ was used to measure paraphilic arousal and behaviors. In the first half, paraphilic interests are measured through 40 items, whereby participants rate their level of sexual arousal on a 7-point Likert scale ranging from *very repulsive* (1) to *indifferent* (4) to *very arousing* (7). The next 40 items relate to engagement in paraphilic activities, whereby participants are asked to rate on a 5-point Likert scale how frequently they have engaged in particular sexual activities, ranging from *never* (1) to *once a year or more on average* (3) to *once a week or more on average* (5). Items on this scale relate to specific paraphilias, such as pedophilia (e.g., “You are having sex with a boy/girl below the age of 12”), fetishism (e.g., “you are kissing, fondling and touching someone’s feet”) and sexual sadism (e.g., “you are forcing someone into sexual activity”). Higher scores on the first 40 items of the Paraphilias Scale represented greater paraphilic interest, whereas higher scores on the other 40 items of the scale indicated increased engagement in sexually deviant behaviors^64^.

### Stimuli and procedure

#### Go/No-Go task

Impulsivity was measured behaviorally by the Go/No-Go Task, using response inhibition as an indication of impulsivity. During the Go/No-Go Task, various stimuli were presented to the participants. The stimuli consisted of different vowels (i.e. ‘a’, ‘e’, ‘i’, ‘o’ and ‘u’), and participants were instructed to rapidly respond when one of the vowels was presented (Go-condition). However, if a vowel was presented two or three times in a row, the participant was instructed to withhold their response (No-Go condition). Overall, 400 trials (275 Go trials, 125 No-Go trials) were presented for 200 ms, with an inter-stimulus-interval (ISI) between 1020 and 1220 ms.

#### Procedure

The study took place physically at the (*location removed for blind review*). Participants received an information letter about the study and the procedure of the experiment. All participants gave informed consent for participation in the study. Participants then received a participant number to link their survey data with their EEG data. Next, participants completed questionnaires on the computer. Participants were then attached to the EEG-device and seated in a comfortable chair in a sound-attenuated EEG room. The participants sat approximately 80–100 cm in front of a 22-inch computer monitor on which the stimuli were presented via E-prime software (Version 2.0; Psychology Software Tools, Pittsburgh, PA). Prior to the experimental tasks, instructions were provided both orally by the experimenter as well as in a written form via an instruction screen on the computer.

Subsequently, participants performed the Go/No-Go task. During the task, the lights in the room were fully dimmed to reduce the influence of distractions. After the experiment, the electrodes were removed, and the participant was debriefed. The duration of the experimental session was approximately 1.5–2 h.

The study has been performed in accordance with the Declaration of Helsinki and received ethical approval from Research Ethics Review Committee of the Erasmus School of Social and Behavioural Sciences of the (*location removed for blind review*) (ETH2122-0208).

#### Electroencephalogram acquisition and analyses

Along with the Go/No-Go Task, an EEG was conducted whereby the ERP component P3 at the CZ electrode was used to measure response inhibition. EEG has a high temporal resolution (1 ms), which allows for measuring early stages of complex, dynamic neural processes related to cognitive control^65^.

EEG signals were recorded using a Biosemi ActiveTwo amplifier system (Biosemi, Amsterdam, The Netherlands). According to the 10–20 International System, 32 active Ag/AgCl electrodes, mounted in an elastic cap, were placed on the scalp. Two additional pairs of electrodes were attached to the left and right mastoids (for referencing), the outer canthi of both eyes (for recording horizontal electro-ocular activity), and the infraorbital and supraorbital region of the left eye (for recording vertical electro-ocular activity).

The recorded raw EEG signals were processed and analyzed offline using Brain Vision Analyzer 2.0 (Brain Products GmbH, Munich, Germany). The EEG signals were filtered using phase shift-free Butterworth filters with a bandpass of 0.10–30-Hz (48 dB). The signal was re-referenced to the average of the left and right mastoid electrodes (M1, M2). Then, EEG data (both for the Go- and No-Go-trials of the Go/No-Go task separately) were segmented in epochs from 200 ms before stimulus presentation to 1000 ms after stimulus presentation. Next, the signal was corrected for ocular movements and blinks using the Gratton and Coles algorithm^66^. Last, epochs exceeding ± 75 mV from the average were presumed to reflect trials with artefact activity and therefore were excluded at any channel from the analysis. Two participants in total were excluded from the ERP analyses, because they had less than ten artefact-free epochs in one of the conditions.

**Figure 2:**
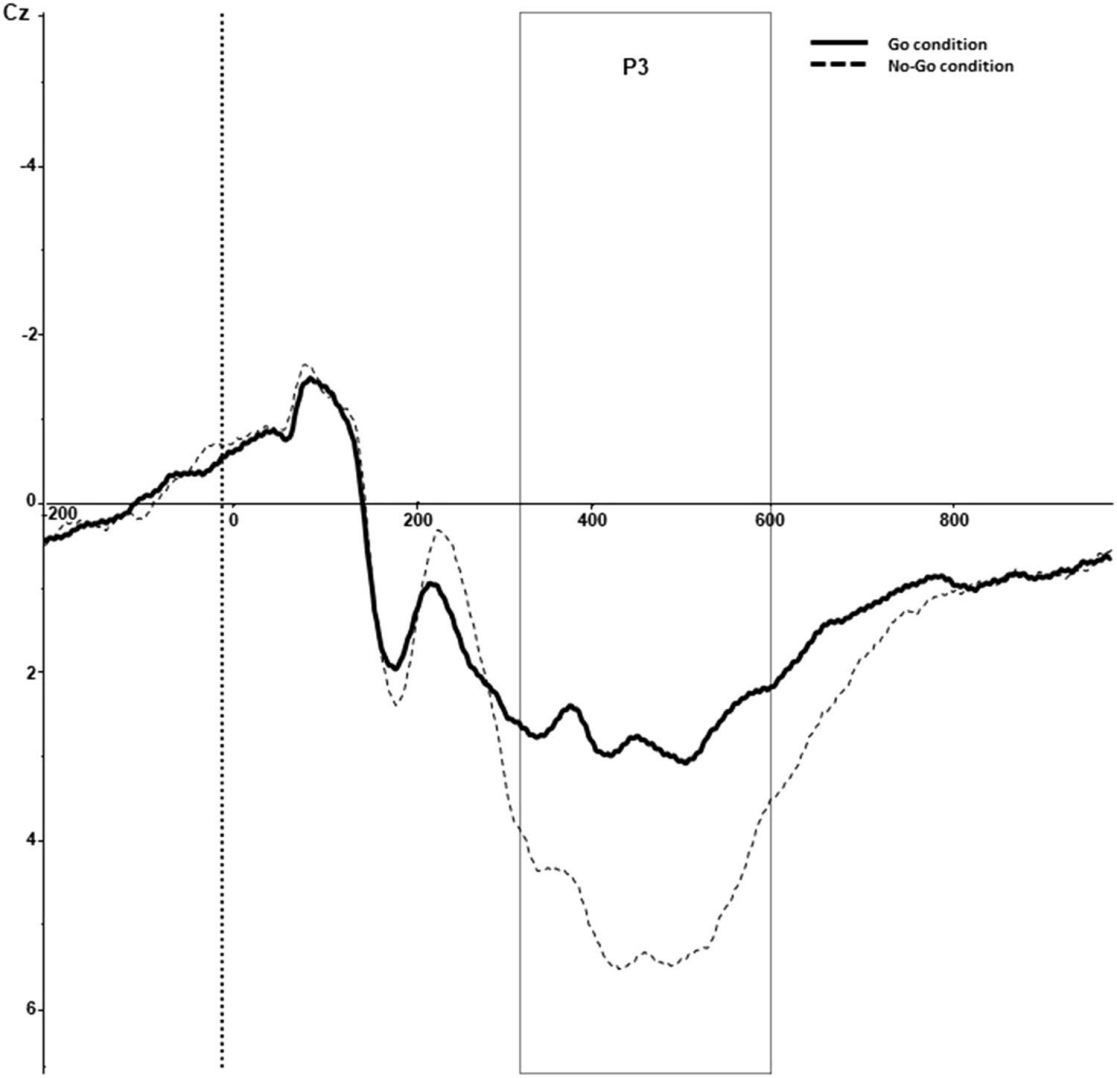
Grand averaged P3 of Go and No-Go conditions at Cz site. Go condition is depicted by a solid black line. No-Go condition is depicted by a dotted black line.

The P3 was defined as the difference score between the average P3 amplitude on Go versus No-Go trials within 300–600 ms after stimulus onset. The average P3 waves were calculated for artefact free trials and compared across the Cz site, as the P3 component is typically observed at midline electrodes during Go/No-Go tasks^67^. Figure 2 depicts the grand-average ERP, including the P3 elicited during the Go/No-Go task. As can be observed from Fig. 2, the P3 amplitude was larger during No-Go-conditions compared to Go-conditions, whereby maximum amplitude peaks were observed around 450 ms at Cz.

## Data Availability

The datasets used and/or analyzed during the current study available from the corresponding author on reasonable request.
